# The Bean Beetle Microbiome Project: A Course-Based Undergraduate Research Experience in Microbiology

**DOI:** 10.3389/fmicb.2020.577621

**Published:** 2020-09-15

**Authors:** Anna J. Zelaya, Nicole M. Gerardo, Lawrence S. Blumer, Christopher W. Beck

**Affiliations:** ^1^Department of Biology, Emory University, Atlanta, GA, United States; ^2^Department of Biology, Morehouse College, Atlanta, GA, United States

**Keywords:** scientific teaching, undergraduate education, course-based undergraduate research, insect microbiomes, 16S rRNA gene sequencing, microbial community analysis

## Abstract

Course-based undergraduate research experiences (CUREs) are an effective means of transforming the learning and teaching of science by involving students in the scientific process. The potential importance of the microbiome in shaping both environmental health and disease makes investigations of microbiomes an excellent teaching tool for undergraduate microbiology. Here, we present a CURE based on the microbiome of the bean beetle (*Callosobruchus maculatus*), a model system for undergraduate laboratory education. Despite the extensive research literature on bean beetles, little is known about their microbiome, making them an ideal system for a discovery-based CURE. In the CURE, students acquire microbiological technical skills by characterizing both culturable and unculturable members of the beetle gut-microbial community. Students plate beetle gut homogenates on different media, describe the colonies that are formed to estimate taxonomic diversity, extract DNA from colonies of interest, PCR amplify the16S rRNA gene for Sanger sequencing, and use the NCBI-nBLAST database to taxonomically classify sequences. Additionally, students extract total DNA from beetle gut homogenates for high-throughput paired-end sequencing and perform bioinformatic and statistical analyses of bacterial communities using a combination of open-access data processing software. Each activity allows students to engage with studies of microbiomes in a real-world context, to apply concepts and laboratory techniques to investigate either student or faculty-driven research questions, and to gain valuable experiences working with large high-throughput datasets. The CURE is designed such that it can be implemented over either 6-weeks (half semester) or 12-weeks (full semester), allowing for flexibility within the curriculum. Furthermore, student-generated data from the CURE (including bacterial colony phenotypic data, full-length 16S rRNA gene sequences from cultured isolates, and bacterial community sequences from gut homogenates) has been compiled in a continuously curated open-access database on the Bean Beetle Microbiome Project website, facilitating the generation of broader research questions across laboratory classrooms.

## Introduction

In recent years, course-based undergraduate research experiences (CUREs) have gained widespread attention as being effective alternatives to “cookbook” style teaching approaches in science, technology, engineering and mathematics (STEM) (Corwin Auchincloss et al., 2014). CUREs allow students to actively participate in the scientific process, experience discovery, do broadly relevant work, practice collaboration, and build on their growing knowledge and experience via iteration (Corwin Auchincloss et al., 2014). By modifying the formal curriculum to integrate an authentic research experience into the classroom, CUREs are able to offer a greater number of students the educational benefits of traditional research experiences (i.e., internships or apprenticeships in faculty research labs) (National Academies of Sciences, Engineering, and Medicine, 2017). These benefits include greater scientific self-efficacy, improved critical thinking skills, increased grades, increased interest in science careers, and higher graduation and retention rates (Lopatto, 2004; Seymour et al., 2004; Corwin et al., 2015a; Staub et al., 2016).

Interdisciplinary research on microbiomes, or the collection of microorganisms and their genes within a given environment, has uncovered the vast diversity and complexity of the microbial world. Microorganisms and their activities are essential for maintaining both human and environmental health (Timmis et al., 2019). In both plants and animals (including humans), microbes assist in the breakdown and absorption of essential nutrients and minerals (Nicholson et al., 2005; van der Heijden et al., 2008) and the development of immunity (Bäckhed et al., 2012). Environmental microbes residing in soil and water recycle nutrients on a global scale (Chapelle, 2000), degrade and transform toxins (Anderson and Lovley, 1997), and contribute toward the removal of greenhouse gases from the atmosphere (Mitchell et al., 2010). Given their broad relevance in shaping the health of our world, microbiomes provide an excellent and engaging topic for undergraduate students and ample opportunities for discovery-based research (Wang, 2017).

Despite the educational potential for laboratory classrooms, microbiome research can be challenging. Many biological systems are highly complex, involving a diverse array of microbes with confounding variation both within and between populations (Spor et al., 2011; Linnenbrink et al., 2013; Goodrich et al., 2014). Disentangling the many genetic and environmental factors shaping this variation is challenging, particularly in organisms that are not easy to control experimentally. Many insect species have served, and continue to serve, as tractable systems for studying the potential factors that shape host-microbe associations (Engel and Moran, 2013; Douglas, 2019). Insect microbiomes tend to be simpler than their vertebrate microbiome counterparts (Wong et al., 2011; Hammer et al., 2017). Additionally, insects can be reared under controlled, laboratory conditions in large numbers, facilitating precise manipulation of environmental factors that may influence microbial associations.

Bean beetles (*Callosobruchus maculatus*) are a tractable insect model system that has been used widely in inquiry-based laboratory education. Bean beetles are inexpensive, commercially available, reproduce rapidly and in large numbers, develop quickly, and are easy to rear and maintain in a classroom environment (Beck and Blumer, 2007). Bean beetles also have broad global relevance, as they are a stored-product pest of dried beans and cause significant economic damage world-wide (Tuda et al., 2006). Although bean beetles have been extensively studied in a wide range of biological disciplines (Beck and Blumer, 2007), very little is known about their microbiome, making them an ideal system for a discovery-based CURE in microbiology. Bean beetles are easily amenable to microbiome experimentation, allowing student researchers to manipulate factors that might affect gut bacteria in ways that would not be possible with vertebrates or other insect model systems.

The Bean Beetle Microbiome Project is a large-scale, multi-year STEM-Education research collaboration with the overarching goal of understanding the role of student autonomy in using scientific practices in a discovery CURE for students across diverse institutions. Here, we present and describe the course materials developed for the Bean Beetle Microbiome CURE that can serve as an inquiry-based course curriculum for undergraduate microbiology laboratories. We also present preliminary survey data from upper-level undergraduates at three different institutions who participated in the first set of implementations of the BBMP-CURE during the Fall 2019 academic term. In the BBMP-CURE, students integrate microbiological, molecular, and bioinformatic techniques to characterize both culturable and unculturable members of the beetle gut-microbial community. We discuss how the CURE can be implemented in either 6-week (half semester) or 12-week (full semester) versions, allowing flexibility within the curriculum. The 6-week format can also be implemented by instructors within institutions that follow the quarter-system. Depending on the research interests of the faculty, many aspects of the CURE outlined below can be easily modified such that students and instructors can take greater ownership of the research questions asked, methods used, and overall research experience. Student-generated data may contribute to ongoing faculty research that subsequently leads to publications. Additionally, faculty have the option of sharing their classroom data within a curated open-access database on the Bean Beetle Microbiome Project website, facilitating the generation of broader research questions across laboratory classrooms and institutions.

## Materials and Equipment

### Learning Objectives

The activities below are designed for students to expand on laboratory skills that they may have practiced in previous introductory laboratory courses (e.g., pipetting, culturing bacteria, and record keeping). However, activities can be easily amended for an introductory class such that basic laboratory skills can be introduced at the time of or prior to performing the activity.

The overall learning objectives for the CURE are for students to be able to:

(1) Describe the impact of microbes on our living planet and their host environments.
(2) Sample and compare microbial communities (microbiomes).
(3) Formulate testable hypotheses to address a research question and design an experiment to test the hypotheses.
(4) Identify and apply the microbiological, molecular, and bioinformatic techniques used to study microbiome data.
(5) Analyze 16S rRNA gene sequence data with common techniques used in microbiome research.
(6) Interpret figures generated from microbiome sequence data.
(7) Communicate findings to peers via oral or poster presentations and scientific writing (report).

To facilitate project ownership, an additional learning objective may be for students to “Discuss and pose a meaningful research question that builds on prior research.” Learning objectives can be modified and amended by the faculty based on the specific research questions asked. Published example rubrics for evaluating student presentations are available (e.g., Kishbaugh et al., 2012).

### Beetle Rearing and Maintenance

**FIGURE 1:**
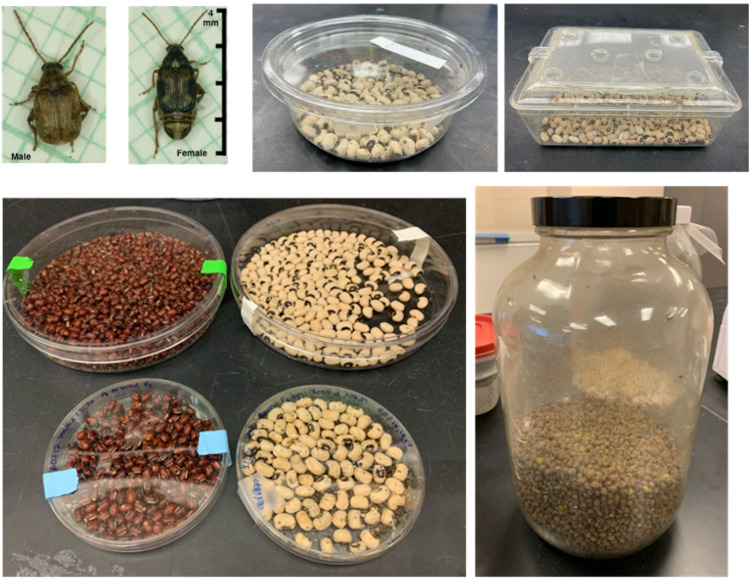
Maintaining bean beetles in the laboratory classroom. Adult (**A**-male, **B**-female) beetles range from about 2–4 mm in length. Beetles cultures can be maintained in a variety of containers filled with dried beans. **(C)** Small round plastic container with lid. **(D)** Plastic bin with lid, holes have been drilled into the lid and lined with filter mesh to allow for gas circulation. **(E)** Petri dishes of various sizes filled with either adzuki beans or black-eyed peas. **(F)** A large glass jar filled with mung beans.

A living culture of bean beetles may be purchased from Carolina Biological Supply Company (Burlington, NC, United States, item number 144180) or Ward’s Science (Rochester, NY, United States, item number 470163-616). Beetles are easily maintained on a countertop in small jars or containers with dried beans (Figure 1). Bean beetles prefer and grow best on black-eyed peas (*Vigna unguiculata unguiculata*); however, beetles grow well on at least eight different bean hosts (see Laboratory Methods at beanbeetles.org), and beans chosen can vary based on the experimental questions to be addressed. Female beetles glue their eggs to the surface of beans. Approximately 8–10 days after oviposition, the beetle larva “hatches” from the egg by burrowing from the egg into the bean, where the developing larva feeds off the bean endosperm. When stored at 25–30°C and developed on black-eyed peas, an adult beetle emerges from the host bean after approximately 25–35 days and becomes sexually mature 24–36 h after emergence. Larval development times may vary depending on host-bean species and environmental conditions (e.g., incubating temperature and humidity). Adult beetles do not eat or drink liquid water, and adults live for approximately 10–14 days. Currently, no U.S. federally mandated permits or requirements are needed to house and use bean beetles for educational or research purposes. Individual U.S. State Department of Agriculture requirements remain in force. The authors advise that educators verify with their home institutions to ensure that no local or institutional requirements exist.

While rearing beetles for classroom activities, it is important to plan accordingly to synchronize emergence times with experiments. Beetle cultures should be grown for a few months in consistent laboratory conditions on the same bean species so as to accurately predict emergence times. Additionally, maintaining replicate lines (on the same bean-host) that have alternating emergence periods can ensure a consistent supply of adult beetles. More details on working with bean beetles can be found at https://www.beanbeetles.org/handbook/.

### Equipment, Supplies, and Reagents

A current list of all equipment, supplies, and reagents used in the different modules of this CURE can be downloaded at https://www.beanbeetles.org/new_website/wp-content/uploads/2019/08/Combined-Equipment-and-Supplies-List-1.pdf. The CURE is designed to use common laboratory equipment available in general microbiology and molecular biology laboratories.

For the bioinformatic analysis portion of the CURE, we designed the activities to utilize free, web-based software that does not require advanced computer science skills (e.g., command-line usage or coding skills). Access to a computer lab would facilitate the implementation of the bioinformatical analysis activities; however, students also may use their personal laptop computers while accessing a university internet connection. Alternatively, bioinformatic analysis may be designated as outside laboratory assignments, allowing students more flexibility to access the technology (computer, internet-connection) necessary to complete the analysis. For example, in Spring 2020, we conducted bioinformatic analyses with undergraduate students in a completely on-line distance learning format.

For taxonomic identification of isolated colonies from beetle gut-homogenates, The Basic Local Alignment Search Tool (BLAST)^1^ is used to identify closest bacterial relatives. For whole community microbiome analysis, spreadsheets containing metadata are created in either Microsoft Excel (Office 365, 2019) or Google Sheets. Bioinformatic analyses are conducted using DNA Subway, which was developed by the DNA Learning Center at Cold Spring Harbor Laboratory. It is a streamlined, classroom-friendly version of the popular QIIME2 microbiome bioinformatics software (Bolyen et al., 2019) and is available via CyVerse^2^, a free-to-use cyber-infrastructure funded by the National Science Foundation that provides computational resources to researchers and educators nationwide (Goff et al., 2011; Merchant et al., 2016). Detailed protocols for all bioinformatics activities are available at https://www.beanbeetles and https://org/microbiome/resources-for-online-teaching/.

### DNA Sequencing

The lessons presented here make use of both Sanger and Next-Generation Sequencing technology in order to provide students a greater breadth of understanding of the tools available to study both cultivable and non-cultivable bacteria at the genetic level (single colony/species vs. whole communities). Currently, the cost for 16S V4 Miseq amplicon sequencing ranges from $55 to $85/sample depending on the number of samples in a single run and whether you use a commercial or academic institution sequencing facility. A meaningful microbiome experiment will likely require a minimum of five samples in each of two treatments, or a total of 10 samples, so whole community sequencing will cost between $550 and $850/class. The cost of NGS-technology can be prohibitive (Wang, 2017). Colleges and universities that cannot secure funding for 16S whole community sequencing may wish to use sample data sets available at https://www.beanbeetles.org/microbiome/resources-for-online-teaching/. As this CURE also incorporates Sanger sequencing of cultivable bacteria, instructors have the option of performing a proxy for the whole community analysis (from NGS technology) with a class-wide Sanger sequencing dataset as a lower-cost alternative (see Student handout on community analysis of colony-based sequence data for this alternate activity).

### Classroom Size and Considerations

The CURE outline below was designed for a laboratory course meeting once a week for a minimum of 3 h. The outlined materials and procedures can accommodate a laboratory of 24 students. Activities were designed for students to work as collaborative teams (e.g., 6 groups of 4 students each). Notably, while the BBMP-CURE was originally designed for hands-on laboratory instruction, much of the components, particularly the modules relating to sequence and community analysis, can be performed in fully virtual-classroom formats.

### Student and Instructor Handouts

All handouts for each laboratory session can be found at https://www.beanbeetles.org/workshops/curriculum-materials/. Instructor handouts provide additional information for preparation of materials. Student handouts provide step-by-step protocols for activities to be carried out for each lesson.

## Methods

The BBMP-CURE has the flexibility of being carried out in either full or half-semester implementations. Both full- and half-semester timelines can be found in Supplementary Tables S1,S2, respectively. The timelines also contain a list of handouts and instructional materials related to weekly activities. These materials can also be found at https://www.beanbeetles.org/workshops/curriculum-materials/. Timelines are constructed assuming a laboratory course that meets once a week for at least 3 h. All protocols and sample datasets can be downloaded at https://www.beanbeetles.org/microbiome/resources-for-online-teaching/.

### Full-Semester Implementations

The first class period serves as an introduction to microbiome research in general. The introduction includes a discussion of how insect microbiomes can be used as research models. An introduction into bean beetles as the model organism provides information on bean beetle ecology, sex-based identification, life-cycle, and agricultural impact. Additionally, students are asked to read published studies relating to bean beetles and microbiome research in various biological systems. Background reading can be assigned as either as a pre-lab or in-class assignments (see suggested reading materials).

The introductory lecture and reading assignments provides the background information and context to facilitate the development of a research question, hypothesis, and experimental design, including identification of control versus experimental groups. The BBMP-CURE uses a guided-inquiry approach to guide students through these initial stages of the project. The research question to be studied is either provided by the instructor (faculty-driven research question), or it is generated by the students themselves through an iterative process of discussion and feedback. Regardless of how the research question is generated (either by the students or by the instructor), students are expected to predict the outcome of the study (generate a hypothesis), as well as to design and conduct the experiment to test their hypothesis, including identifying the control versus experimental groups. Instructors may choose to structure a more faculty-driven or student-driven approach. The approach taken may depend on student level (e.g., freshman versus senior), course type (e.g., introductory vs. advanced courses), or course objectives. Additionally, the instructor may choose to have students formulate their research question(s) based on class consensus, or alternatively, if the budget permits, different groups may choose to answer different questions. Example research questions that have been generated by students of the BBMP-CURE using a guided-inquiry approach can be found in Supplementary Table S3.

By week 2, students and instructors have agreed upon the research question(s) to be investigated. Subsequently, students extract DNA from beetle gut homogenates for whole-community sequencing. The timing of DNA extraction depends on the nature of the experiment being conducted. Comparisons of microbiomes from individuals taken from mass cultures (for example, comparing the microbiomes of males and female beetles) may be performed as early as week 2 or 3 if those cultures were previously established. However, a manipulation experiment will require three weeks or longer for the development and emergence of adults (for example, the effect of a host shift on the microbiome community). In classroom settings, waiting for adult emergence is necessary, as larvae are embedded in beans and may be hard for students to successfully isolate without contamination. The steps outlined below describe DNA extraction using the Qiagen DNeasy Blood and Tissue Kit. Qiagen DNeasy Blood and Tissue kit was chosen for this module based on previous literature that demonstrates its successful retrieval of insect-microbiome DNA (Furgeson et al., 2018).

DNA is extracted using a modified protocol for the purification of total DNA from insects (see both Instructor and Student Handouts on DNA extraction for the full semester implementation^3^). Prior to extraction, live adult beetles must first be transferred to a small sterile container (e.g., Petri-dish or microcentrifuge tube) and sacrificed by freezing (−20°C) for a brief period of time (e.g., 3-min). Students can perform the freezing step, or alternatively instructors may sacrifice the beetles before the start of the class session. Once students receive their sacrificed beetles, they should record relevant specimen information (e.g., host diet, sex, age, etc.) prior to surface sterilization. Then, surface-sterilized beetles are transferred to a 1.5 mL microcentrifuge tube containing 180 uL of Buffer ATL. Using a sterile disposable pestle, beetles are crushed to release microbial cells from the beetle gut. The extracted DNA can be quantified (e.g., with a Nanodrop), and stored at −20°C until sequencing.

It is not uncommon for students to extract insufficient DNA on their first attempt. Therefore, week 3 of the full-semester implementation has been designated as an iteration day, allowing students the opportunity for a second attempt at DNA extraction. Students can repeat unsuccessful extractions, or students who successfully extracted sufficient DNA in week 2 can use the time to isolate DNA from an individual from another treatment group to increase sample size. Alternatively students who do not need to repeat an extraction can proceed to culture bacteria from gut homogenates (see below).

The DNA extraction step is ideally performed early in the semester (week 3) in order to provide ample time for sequencing to be performed. Although typical turnaround times for paired-end whole community sequencing can vary, we have allotted ∼4 weeks from the time the DNA is sent out to retrieval of sequence data for community analysis.

Once DNA extractions have been performed, students initiate culture-based analysis of microbes isolated from beetle gut-homogenates. Beetles are surface sterilized and transferred to a 1.5 mL microcentrifuge tube containing 500 uL of 0.9% sterile saline solution. Each beetle is crushed with a sterile pestle and cell-homogenates are serially diluted and plated on different solid-media to obtain bacterial cultures from the beetle microbiome. The current module outlines the use of a general growth media (nutrient agar) as well as the use of selective media for Gram-positive (phenyl-ethyl alcohol) and Gram-negative (eosin-methylene blue) bacteria. Students allow their plates to grow at room temperature (25°C) until the following class period. However, the instructors may opt to utilize any number of general or selective medias to appropriately pursue the specific research question being addressed by their students. Additionally, initial growth in liquid cultures can be used to pursue questions related to physiological or metabolic capabilities of the beetle microbiome.

The following class period (week 4), students check the growth of their microbial cultures. They observe and record the phenotypic characteristics of grown colonies. Using a colony-based PCR approach, students then amplify DNA from specific colonies of interest for taxonomic identification via Sanger sequencing of the 16S rRNA gene (see both Instructor and Student Handouts on colony-based PCR). Here it is important to save students’ plates, as the following week will allow for iteration (a second attempt at PCR for any groups who do not obtain a successful amplified product). Additionally, students who took advantage of the iteration for DNA extraction in week 3 may catch-up this week by crushing beetles and plating the gut-homogenates onto solid-media.

In week 5, students who performed colony-based PCR in week 4 visualize their amplified products using gel electrophoresis. Additionally, week 5 can serve as an iteration day for students who may still need to catch up (either they need to perform colony-based PCR on their isolates for the first time, or their PCRs did not yield enough DNA and they need to redo their PCR). All successfully amplified DNA products should then be sent out for Sanger sequencing.

As Sanger-sequencing turn around is typically fast (∼24–48 h), students should have their Sanger-sequence data by the following class period (see Student Handout on BLAST analysis of sequencing data). Students use the NCBI-nBLAST database to taxonomically classify the colony-based PCR sequences, and create a taxonomy table of the bacterial genera identified in the microbiomes of the beetles in their experiment. This class session serves as an introduction to bioinformatics tools and computational software to compare genetic sequences for taxonomic relatedness. The concepts learned in these sessions will continue to be built upon as they proceed through the semester.

In week 7, students are introduced to community analysis. They are first introduced to this concept by performing community analysis on the phenotypic data that they previously collected on microbes isolated on solid-media. They connect the phenotypic data from week 4 with the colony-based PCR sequence data from Sanger sequencing. They calculate alpha-diversity statistics based on the taxonomy table of different taxa identified from the colony-based PCR sequence data and determine similarities and differences in diversity between samples (Blumer and Beck, 2020). The worksheet for these activities can be downloaded in the course materials for week 7 of the full semester.

By week 8, the whole community (paired-end) sequence data should be available. These data permit students to expand on the community analysis by identifying bacterial taxa in the whole-community dataset using DNA Subway. Weeks 9 through 13 are then reserved for bioinformatic analysis of microbial community sequence data. Since this is the most challenging technical aspect of the CURE, several sessions are allotted to complete this activity. Multiple options are available here to cater to varying degrees of experience and pre-requisite knowledge of students and instructor. These include community analysis using the R Statistical Package, spreadsheets in Excel or Google Sheets, or the free web-based Shiny App ranacapa (Kandlikar et al., 2018). Alternatively, the semester can be modified such that activities are assigned as outside classwork (homework), allowing for greater flexibility of the curriculum.

In the final week, students develop their scientific communication skills by presenting their results. Instructors may choose presentation methods to match their course learning objectives (scientific abstract, full scientific report, seminar style presentation, poster presentation). Undergraduates may also take advantage of opportunities to present research outside of the classroom at their home institutions (for example, a departmental undergraduate research symposium or college-wide science symposium).

### Half-Semester Implementations

Similar to the full-semester implementation, the first class period for the half-semester implementation serves as an introduction to insect microbiomes in general and bean beetles as the model organism. Additionally, the research question and experimental design should be discussed and agreed upon during the first class session. However, the introduction period is shortened such that students initiate their research project in the first class period. Once students and instructors agreed on the research question(s) to be investigated, students are provided with their specimen beetles and follow surface sterilization and homogenization steps to plate beetle-gut homogenates onto general and selective media.

The following class period (Week 2), students check the growth of their microbial cultures, observe and record the morphological characteristics of grown colonies, and perform colony-based PCR on their isolated colonies for taxonomic identification via Sanger sequencing (see both Instructor and Student Handouts on colony-based PCR for the half-semester implementation^3^). Again, all plates can be saved for the following week in case students require an iteration day. Finally, students obtain a fresh collection of beetles to perform total DNA extraction for whole-community sequencing using the Qiagen DNeasy Blood and Tissue Kit. Completing DNA extraction may require students to perform laboratory work outside of scheduled class time, or for someone to perform these extractions after students initiate the process by grinding a beetle in Buffer ATL (see both Instructor and Student Handouts on DNA extraction for the half-semester implementation^3^).

In week 3, students perform gel-electrophoresis on their amplified DNA (see Instructor handouts on electrophoresis). Typically, electrophoresis can be completed in about 1 h, at which point any groups that did not successfully amplify DNA may elect to re-do their PCR on a picked colony. Then, successfully amplified DNA products may be sent out for Sanger sequencing. In a typical 3-h laboratory course, students would have enough time to run a gel and re-do PCR for any unsuccessful amplifications. However, running a gel on the second PCR attempt to confirm amplification may require students to perform laboratory work outside of scheduled class time.

The following class periods (weeks 4, 5, and 6), students begin the bioinformatic analyses. They use the NCBI-nBLAST database to taxonomically classify sequences (see Student handout on BLAST analysis of sequencing data), perform community analysis on phenotype and colony-based PCR sequence data (see student handout on community analysis of phenotype data), and perform community analysis of their whole microbiome sequencing data (see handouts corresponding to community analysis of sequencing data for week 6 of the half-semester implementation). The half-semester implementation allows insufficient time for students to learn to use DNA Subway to create a taxonomy table for subsequent community ecology analysis. Therefore, it is necessary for the instructor or teaching assistant to perform the data processing in DNA Subway and present students with the resulting taxonomy table for analysis. As in the full-semester implementations, students can present their results in the final week of the project in either the written or oral formats suggested above.

## Anticipated Results and Discussion

Timmis et al. (2019) recently argued that microbiology literacy is one of the most important skills society will need to solve 21^st^ century problems. Before long, novel therapies on microbiome technology will become widely available (Surana, 2019). However, misconceptions and misinformation persists (Ma et al., 2018). Undergraduate university courses represent an excellent opportunity to introduce students of microbiology to the exciting and topically relevant field of microbiome research, as they represent the future leaders and global citizens that will be best equipped to dispel misconceptions and raise microbial literacy among the general public.

While still in its early stages, microbiome research has grown rapidly and garnered the attention of both scientists and non-scientists alike, providing an excellent and engaging topic area for undergraduate research experiences. Inquiry-based activities for student microbiome research have previously been developed for aquatic ecosystems (Boomer et al., 2002; Gibbens et al., 2015; Agate et al., 2016), soil (Martinez-Vaz et al., 2015; Finer et al., 2016; Rahman et al., 2016), public spaces (Muth and McEntee, 2014; Weber and Werth, 2015), and the human microbiome (Wang et al., 2015; Debelius et al., 2016; Garbarino and Mason, 2016; Lentz et al., 2017; Weber et al., 2018). Here we present a CURE using the bean beetle (*Callosobruchus maculatus*) as a model animal system for undergraduate microbiology laboratory courses.

The Bean Beetle Microbiome CURE is aimed to provide students with an authentic research experience as part of the course curriculum that enables them to develop skills related to the scientific process. Studies have shown that research experiences provide students with a greater sense of autonomy and ownership of their projects (Lopatto, 2003; Hanauer et al., 2012), which also leads to additional educational benefits, such as increased intention to pursue science careers. When research experiences are incorporated as part of the course curriculum, there are different inquiry-based approaches that can be used, each with its own set of expectations for both instructor and student (D’Avanzo, 1996; Colburn, 2000; Buck et al., 2008; Weaver et al., 2008). Faculty who would like to implement the CURE presented here in their laboratory classrooms may choose the degree of student involvement for deciding the research question and what inquiry-based approach to use. The novelty and relevance of the question addressed may depend on several factors, including the educational level of the student (e.g., introductory vs. advanced) as well as the level to which the research design is guided by faculty.

An important component of our current research on the BBMP-CURE is the level of student autonomy, or the level of responsibility, related to the development of the research question addressed. Our approach provides two levels of autonomy that we categorize as either low-autonomy (faculty-driven questions) or high-autonomy (student-driven questions). While we hypothesize that a greater level of student autonomy will have a positive effect on student outcomes, it is anticipated that students will strengthen their science-process skills regardless of the level of autonomy chosen, as they analyze the results of their experiments, draw conclusions, connect their research to the broader literature, and communicate their results. Our ongoing research based on assessments of survey data for past and future implementations of the BBMP-CURE will be used to test this hypothesis.

In the fall and spring semesters of the 2019–2020 academic year, a total of 10 faculty participants from six different institutions (most of which were minority-serving institutions) implemented the BBMP-CURE (Supplementary Figure S1A). Due to the interdisciplinary nature of the CURE, it was successfully implemented in undergraduate laboratories of various life-science disciplines and student class-levels (Supplementary Figures S1B,C). Students who participated in the BBMP-CURE were asked to respond to questions from the Persistence in the Sciences Survey (PITS) (Hanauer et al., 2016) and the Laboratory Course Assessment Survey (LCAS) (Corwin et al., 2015b). As our study did not include a “traditional lab” control, we included two previously published studies that used these same instruments to assess inquiry-based versus traditional laboratory instruction as benchmarks for comparison (Corwin et al., 2015b; Hanauer et al., 2017). Our preliminary student survey data on the LCAS (Supplementary Table S4) shows that students rated the BBMP-CURE highly on the Discovery and Relevance scale (28.2 ± 6.3 Full-Semester/Low Autonomy; 28.88 ± 1.81 Full-Semester/High Autonomy; 23.5 ± 11.83 Half-Semester/Low Autonomy), suggesting that students perceived that the activities they performed could lead to discovery of something new and were of interest to the scientific community. Additionally, results of the PITS survey (Supplementary Table S5) showed students scored highly on questions pertaining to science-identity (3.8 ± 0.8 Full-Semester/Low Autonomy; 4.04 ± 0.77 Full-Semester/High Autonomy; 4.4 ± 0.69 Half-Semester/Low Autonomy), scientific community values (4.9 ± 0.9 Full-Semester/Low Autonomy; 5.11 ± 0.59 Full-Semester/High Autonomy; 5.13 ± 0.88 Half-Semester/Low Autonomy), project ownership of content (4.0 ± 0.6 Full-Semester/Low Autonomy; 3.92 ± 0.6 Full-Semester/High Autonomy; 0.88 ± 5.13 Half-Semester/Low Autonomy), and emotional project ownership (3.8 ± 0.6 Full-Semester/Low Autonomy; 3.78 ± 0.89 Full-Semester/High Autonomy; 4.12 ± 0.6 Half-Semester/Low Autonomy). While these preliminary results are promising, additional data collected from future implementations of this CURE will allow for a more thorough analysis to evaluate the impact of the BBMP-CURE on student outcomes.

Despite the reported educational benefits of CUREs, challenges to their implementation persist (Spell et al., 2014; Cooper and Brownell, 2018). Logistical hurdles and lack of time to develop new laboratory research experiences are often cited as barriers that faculty face when considering a CURE (Spell et al., 2014; Shortlidge et al., 2016). The difficulties can be especially prominent for faculty members who work within primarily undergraduate institutions with limited access to research personnel and resources. The Bean Beetle Microbiome CURE overcomes these barriers as it is flexible, scalable, and utilizes supplies and techniques common for microbiology and biology classrooms. All protocols for the above modules are freely available at https://www.beanbeetles.org/workshops/curriculum-materials/ and https://www.beanbeetles.org/microbiome/resources-for-online-teaching/. Additionally, many of these protocols are also available in the *Proceedings of the Association for Biology Laboratory Education*, with detailed Instructor Notes and implementation guidance (Cole et al., 2018; Blumer and Beck, 2020), although the protocols presented here (and available on our website) offer revised and updated methods and notes based on participant feedback. The CURE has been designed such that educators have the freedom and flexibility to modify activities within the CURE to implement methods and techniques related to their own research interests. For example, during the cultivation of beetle-gut microbiome isolates, activities may be introduced that allow students to perform classical microbiological and microscopic analyses, such as differential staining (e.g., Gram-stain, acid-fast stain, endospore stain, etc.), hemocytometer-based microscopic cell counts of bacteria, and any number of physiological and metabolic assays. Additionally, the potential exists for students to conduct research that aligns with the independent research interests of the faculty. Thus, faculty have the opportunity to replicate studies from previous implementations, allowing future students to build on the findings from previous semesters, which can lead to more publishable work (LoSchiavo, 2018). Students and educators may take advantage of the current database of information on isolated microbes that is available on the Bean Beetle Microbiome Project website (beanbeetles.org), facilitating the generation of broader research questions across laboratory classrooms. Research on the bean beetle microbiome can be further facilitated by the open sharing of student-collected data. Currently, over 490 isolates are publicly available in the colony-based sequence and taxonomy databases at www.beanbeetles.org, and faculty are encouraged to submit their student-collected data to the database. While these data are publicly available, faculty who implement this CURE maintain ownership of the data collected by their students.

Another challenge of CUREs is that the authentic nature of the research means that, like with all research, students likely will carry out procedures that do not work in the first attempt. Research is inherently an iterative process where scientists must navigate scientific challenges, persevere through difficulties, and cope with failure (Henry et al., 2019). It has been argued that, for a research experience to be authentic, opportunities for iteration must be included (Corwin Auchincloss et al., 2014), as they allow for students to develop cognitive and emotional ownership of their work (Corwin et al., 2018). In the full-semester implementation of the Bean Beetle Microbiome CURE, iteration for procedures that commonly require multiple attempts (e.g., DNA extraction, PCR amplification) is built-in to the course schedule. A limitation of the half-semester implementation is that, due to the reduced time for completing the project, many iterative opportunities are not feasible.

The most significant barrier to incorporating microbiome research for undergraduate education is the cost associated with next-generation sequencing (NGS) (Hartman et al., 2016). This CURE outlines the use of the Illumina MiSeq System for paired-end sequencing of the beetle-gut microbiome. As NGS is now a relatively common technology, educators may have access to this or other sequencing platforms via their home universities at “in-house” costs, or may collaborate with colleagues who have access to NGS technology. Additionally, academic and commercial sequencing centers are available throughout the country that provide sequencing services and relatively low per-sample costs. The turn-around time for sequencing and data retrieval may vary, and educators interested in performing the NGS portion of this CURE should plan accordingly such that students have enough time to analyze data. Some sequencing centers provide data analysis for an additional cost, which may be of interest to educators whose desired learning outcomes are related to data interpretation as opposed to data processing. Additionally, the CURE is designed such that if NGS cost is prohibitive, a proxy for community analysis can be performed with phenotypic data of cultured isolates on solid media and colony-based PCR sequence data. Extensive sample datasets for both types of data are available in our open-access databases (Beanbeetles.org).

Other challenges may arise in the sessions dedicated to sequence data analysis using computational methods. Depending on the classroom set up, certain technical difficulties may arise (e.g., slow/unreliable internet connections, students who do not own personal laptops, students with different levels of familiarity with computers or spreadsheets). A teaching assistant or peer-mentor who can move around freely to aid students who may be struggling can be a valuable asset for these laboratory sessions. Additionally, access to university computational resources (e.g., computer labs) may alleviate some of the challenges of performing computational analysis in the laboratory classroom. For the DNA Subway portion of the bioinformatic analysis, students are encouraged to work in groups such that only one computer per group is logged on to the server to perform the metagenomic analysis, thereby reducing demands on the server that might prolong time to completion of the analysis. Alternatively, faculty may opt to have students perform the analyses as outside classwork so that students can have ample time to complete the analyses. Written and video tutorials are available to facilitate students’ independent remote learning^3^. This option also allows for class time to instead be dedicated to interpretation of graphs and data.

Finally, while the BBMP-CURE was originally designed for hands-on laboratory instruction, the global outbreak of COVID-19 has significantly altered current instructional programs. It is uncertain when and to what extent opportunities for in-person instruction will become available again for all undergraduates. The current situation facing universities underscores the need for quality classroom activities that can be performed in online and virtual formats. The work presented here adds value to the current state of instruction by providing easy-to-implement modules with access to our online-database of isolate and whole-community sequence datasets, which allows for virtual implementation of many aspects of this CURE.

## Data Availability Statement

The datasets presented in this study can be found in online repositories. The names of the repository/repositories and accession number(s) can be found in the article/ Supplementary Material.

## Ethics Statement

The studies involving human participants were reviewed and approved by Emory University Institutional Review Board (IRB00113934). The patients/participants provided their written informed consent to participate in this study.

## Author Contributions

AZ performed literature review, created handouts and instructional materials, and wrote and edited majority of the manuscript. NG, LB, and CB created handouts and instructional materials and wrote and edited the manuscript.

## Conflict of Interest

The authors declare that the research was conducted in the absence of any commercial or financial relationships that could be construed as a potential conflict of interest.

## Footnotes

1 http://blast.ncbi.nlm.nih.gov/

2 https://www.cyverse.org

3 https://www.beanbeetles.org/workshops/curriculum-materials/

## Supplementary Material

FIGURE S1 Survey data collected from faculty participants of the Bean Beetle Microbiome Project for the 2019–2020 academic year.

TABLE S1 Course timeline for full-semester implementation.

TABLE S2 Course timeline for half-semester implementation.

TABLE S3 Example student and/or faculty-generated research questions implemented in the 2019–2020 academic year.

TABLE S4 Preliminary results of group differences on the Laboratory Course Activities Survey (LCAS).

TABLE S5 Mean and standard deviation for student responses from the Persistence in the Sciences (PITS) Survey.
