# Supplementary material for: The Bean Beetle Microbiome Project: A Course-Based Undergraduate Research Experience in Microbiology
Source: Front Microbiol. 2020 Sep 15;11:577621. doi: 10.3389/fmicb.2020.577621 (PMC7522406; doi:10.3389/fmicb.2020.577621)
Supplement: FIGURE S1 — Survey data collected from faculty participants of the Bean Beetle Microbiome Project for the 2019–2020 academic year. [file Image_1.PDF]

Supplementary Material

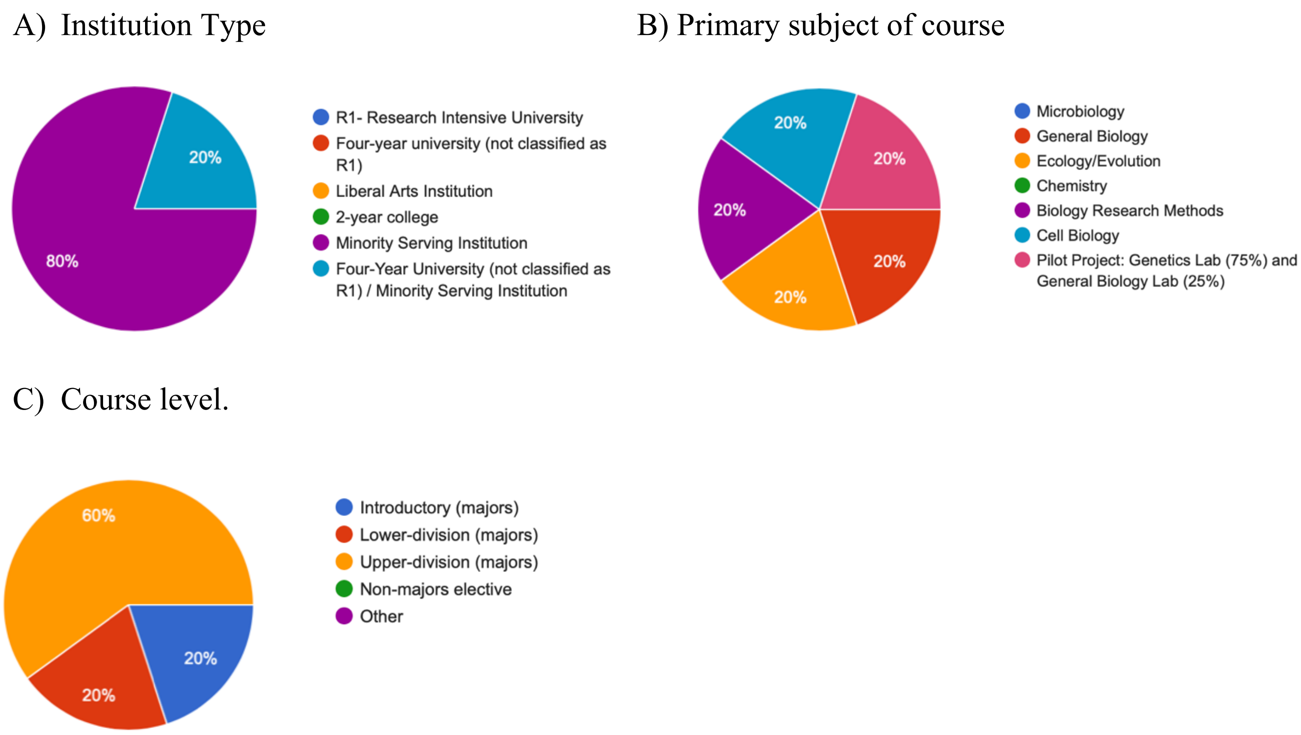

Supplementary Figure 1. Survey data collected from faculty participants of the Bean Beetle Microbiome Project for the 2019-2020 academic year. A total of 10 faculty participants from 6 different institutions implemented the Bean Beetle Microbiome CURE in the 2019-2020 academic year. A brief 12-question survey was emailed to all participating faculty. A total of 5 (N=5) faculty instructors representing 5 different institutions responded to the survey. A) The majority of faculty who responded implemented the BBMP-CURE at minority serving institutions (80%). These data reflect the focus on the recruitment of faculty from minority-serving institutions for this first year of implementation. Upcoming implementations will expand recruitment to 2-year institutions, liberal arts colleges, and research intensive (R1) universities. B) Based on responses, the CURE was successfully implemented in a wide-range of life-science courses. C) Responses indicate that the majority of students who participated in the CURE were upper division students majoring in their discipline (60%).

frontiers
