# Supplementary material for: The Bean Beetle Microbiome Project: A Course-Based Undergraduate Research Experience in Microbiology
Source: Front Microbiol. 2020 Sep 15;11:577621. doi: 10.3389/fmicb.2020.577621 (PMC7522406; doi:10.3389/fmicb.2020.577621)
Supplement: TABLE S1 — Course timeline for full-semester implementation. [file Table_1.docx]

Supplementary Table 1 Full-semester implementation schedule

| Week |  | Instructor Resources |
| --- | --- | --- |
| 1 | Introduction to insect microbiomes, bean beetles, experimental design, & culturing of microbes | - Introduction to Microbiomes presentation slides - Introduction to bean beetles presentation slides - Instructor’s notes on introduction to bean beetles - Instructor’s notes on preparing media & pouring plates - Instructor’s notes on culturing microbial communities - Student handout on culturing microbial communities |
| 2 | DNA extraction for microbial community sequencing | - Instructor’s notes on DNA extraction - Student handout on DNA extraction - DNAeasy Blood & Tissue Insect Protocol |
| 3 | Iteration day for unsuccessful DNA extractions |  |
| 4 | Phenotypic assessment of microbes & culture-based PCR of selected colonies | - Instructor’s notes on culturing microbial communities - Instructor’s notes on colony-based PCR - Student handout on culturing microbial communities - Student handout on colony-based PCR |
| 5 | Electrophoresis and iteration day for unsuccessful culture-based PCR | - Instructor’s notes on electrophoresis - Student handout on colony-based PCR |
| 6 | Introduction to bioinformatics & analysis of colony sequencing data | - Student handout on BLAST analysis of sequencing data |
| 7 | Introduction to community analysis, phenotype & colony sequencing analysis | - Studying microbiomes presentation slides - Instructor handout on community analysis - Student handout on community analysis of phenotype data - Student handout on community analysis of colony-based sequence data |
| 8 | Introduction to DNA Subway and analysis of microbial community sequencing data | - CyVerse Tutorial - DNA Subway Tutorial - DNA Subway Video Tutorial - Creating a metadata file for DNA Subway and Community analysis in R |
| 9-11 | Bioinformatic analysis of microbial community sequencing data | - DNA Subway tutorial - DNA Subway video tutorial |
| 12-13 | Community analysis of sequencing data | - Preparing files for analyses - Community analysis in Excel - Community analysis in Google Sheets - Community analysis in R - Community analysis in ranacapa - Community analysis in ranacapa tutorial |
| 14 | Final presentations |  |
