# Supplementary material for: The Bean Beetle Microbiome Project: A Course-Based Undergraduate Research Experience in Microbiology
Source: Front Microbiol. 2020 Sep 15;11:577621. doi: 10.3389/fmicb.2020.577621 (PMC7522406; doi:10.3389/fmicb.2020.577621)
Supplement: TABLE S3 — Example student and/or faculty-generated research questions implemented in the 2019–2020 academic year. [file Table_3.DOCX]

Supplementary Material

**Supplementary Table 3. Example Student-generated research questions implemented in the 2019-2020 academic year**

Q1. **Can differences in bean beetle gut-microbiome based on sex differences (male vs female)?**

Q2. **Do beetles who feed on different bean species have different microbiomes?**

Q3. **Does altering the pH of bean surfaces affect microbiome composition of bean beetles fed on black eyed peas?**

Q4. **Are there differences between the gut-microbiome of male bean beetles belonging to two different closely related strains? (sub-species?)**

Q5. **Do dead beetles maintain the same microbial community composition as living beetles?**

Q6. **Do microbiomes differ between beetles who feed on sterile vs unsterile beans?**
