# Supplementary material for: The Bean Beetle Microbiome Project: A Course-Based Undergraduate Research Experience in Microbiology
Source: Front Microbiol. 2020 Sep 15;11:577621. doi: 10.3389/fmicb.2020.577621 (PMC7522406; doi:10.3389/fmicb.2020.577621)
Supplement: TABLE S4 — Preliminary results of group differences on the Laboratory Course Activities Survey (LCAS). [file Table_4.DOCX]

Supplementary Material

|  | BBMP Students (Full-Semester, Low Autonomy) | |  |  | BBMP Students (Full Semester, High Autonomy) | |  |  | BBMP Students (Half-Semester, Low Autonomy) | |  |  | CURE Students (LCAS)* | |  |  | Traditional Students (LCAS)* | |  |  | Possible range of scores |
| --- | --- | --- | --- | --- | --- | --- | --- | --- | --- | --- | --- | --- | --- | --- | --- | --- | --- | --- | --- | --- | --- |
|  | Mean | SD | n |  | Mean | SD | n |  | Mean | SD | n |  | Mean | SD | n |  | Mean | SD | n |  |  |
| Collaboration | 20.5 | 5.15 | 28 |  | 20.25 | 6.45 | 8 |  | 18.17 | 7.40 | 12 |  | 21.11 | 3.20 | 73 |  | 20.87 | 4.02 | 68 |  | 6-24 |
| Discovery/Relevance | 28.2 | 6.30 | 29 |  | 28.88 | 1.81 | 8 |  | 23.50 | 11.83 | 11 |  | 24.35 | 4.04 | 72 |  | 20.77 | 5.82 | 61 |  | 5-30 |
| Iteration | 24.9 | 3.53 | 29 |  | 25.63 | 1.51 | 8 |  | 21.08 | 8.40 | 12 |  | 28.71 | 4.15 | 72 |  | 26.53 | 7.00 | 62 |  | 6-36 |
| LCAS Total | 73.6 | 8.60 | 28 |  | 74.75 | 6.65 | 8 |  | 62.75 | 22.55 | 11 |  | 75.10 | 8.67 | 60 |  | 68.15 | 14.76 | 55 |  | 17-90 |

**Supplementary Table 4.** Preliminary results of group differences on the Laboratory Course Activities Survey (LCAS)(Corwin et al., 2015b) for student participants of the BBMP-CURE of the Fall 2019 academic term. Surveys containing questions from the LCAS were created for each class in Qualtrics, and the survey link was sent to students by their instructors. Student participation in the survey was optional and an alternative assignment was given if a student opted out of the survey. The LCAS survey rating scales range from one (strongly disagree) to six (strongly agree) for all measures except for collaboration, which ranged from one (never) to four (weekly) scale. Sum totals of student LCAS ratings for each category were averaged. ^*^Published data from Corwin et al. 2015b are included and serve as a benchmark for which to compare BBMP-CURE results. The student assessment was approved by Emory University’s Institutional Review Board (IRB00113934).
