# Supplementary material for: The Bean Beetle Microbiome Project: A Course-Based Undergraduate Research Experience in Microbiology
Source: Front Microbiol. 2020 Sep 15;11:577621. doi: 10.3389/fmicb.2020.577621 (PMC7522406; doi:10.3389/fmicb.2020.577621)
Supplement: TABLE S5 — Mean and standard deviation for student responses from the Persistence in the Sciences (PITS) Survey. [file Table_5.DOCX]

Supplementary Material

|  | BBMP Students (Full-Semester, Low Autonomy) | |  | BBMP Students (Full Semester, High Autonomy) | |  | BBMP Students (Half-Semester, Low Autonomy) | |  | SEA-PHAGES^*^ | |  | Traditional LAB^*^ | |
| --- | --- | --- | --- | --- | --- | --- | --- | --- | --- | --- | --- | --- | --- | --- |
| sample size (n) | 33 | |  | 9 | |  | 14 | |  | 335 | |  | 104 | |
|  | Mean | SD |  | Mean | SD |  | Mean | SD |  | Mean | SD |  | Mean | SD |
| Self-Efficacy | 4.1 | 0.9 |  | 3.76 | 1.04 |  | 4.36 | 0.61 |  | 4.12 | 0.55 |  | 3.99 | 2.32 |
| Science Identity | 3.8 | 0.8 |  | 4.04 | 0.77 |  | 4.40 | 0.69 |  | 3.90 | 0.73 |  | 3.47 | 0.99 |
| Scientific Community Values | 4.9 | 0.9 |  | 5.11 | 0.59 |  | 5.13 | 0.88 |  | 5.13 | 0.92 |  | 4.76 | 0.99 |
| Project Ownership-Content | 4.0 | 0.6 |  | 3.92 | 0.60 |  | 4.36 | 0.67 |  | 3.96 | 0.55 |  | 3.4 | 0.66 |
| Project Ownership-Emotion | 3.8 | 0.6 |  | 3.78 | 0.89 |  | 4.12 | 0.60 |  | 3.82 | 0.55 |  | 3.32 | 1.32 |
| Networking | 3.2 | 1.0 |  | 2.91 | 0.93 |  | 3.64 | 1.28 |  | 3.74 | 0.92 |  | 3.03 | 0.99 |

**Supplementary Table 4.** Mean and standard deviation for student responses from the Persistence in the Sciences (PITS) Survey (Hanauer et al., 2016) for students who participated in BBMP full-semester, low-autonomy (n=33), full-semester, high autonomy (n=9), and half-semester, low autonomy (n=14). Surveys containing questions from the PITS survey were created for each class in Qualtrics, and the survey link was sent to students by their instructor.  Student participation in the survey was optional, and an alternative assignment was given if they opted out of the survey. The PITS survey rating scales range from one (strongly disagree) to five (strongly agree) for all measures except for scientific community values, which ranged from one (not like me at all) to six (very much like me) scale. ^*^Published data from the Science Education Alliance-Phage Hunters Advancing Genomics and Evolutionary Science (SEA-PHAGES) program (Hanauer et al., 2017) are included and serve as a benchmark for which to compare BBMP-CURE results. The student assessment was approved by Emory University’s Institutional Review Board (IRB00113934).
